# Preliminary Anesthesia as an Aid in the Diagnosis of Fracture—Consultation*Extract from a lecture on closed fractures.

**Published:** 1901-06

**Authors:** Thomas H. Manley

**Affiliations:** New York


### PRELIMINARY ANESTHESIA AS AN AID IN THE DIAGNOSIS OF FRACTURE—CONSULTATION.*

BY THOMAS H. MANLEY, M D.,

NEW YORK.

*Narcotic relaxation* of the muscles we must sometimes resort to in hyperesthetic conditions of the limb in children or hysterical women, or in many obscure or doubtful cases when a diagnosis is urgently called for. When other measures fail, and muscular spasm is extreme, pulmonary-anesthesia carried just far enough to obliterate the pain sense will slacken the grip of the muscles quite enough to allow motion of the locked-up fragments. In some cases a critical precise examination of a doubtful case is impossible without this precious resource. A little over a year ago a case came under my care which proved its great value. A gentleman of forty years sustained a severe injury to his hip-joint from a fall. The family practitioner being in doubt as to the character of the injury, called in one of our best known surgeons for a consultation. It was then decided that the injury was a severe contusion with a resulting arthritis. But the man continued from bad to worse, suffering the greatest torture on any movement of the limb or body. A week after the first consultation the case was seen by me with

* Extract from a lecture on closed fractures.

the doctor. Now, on a strong table he was placed and under an anesthetic, crepitus with dislocation was clearly obvious. The Roentgen Rays all must concede as one of the most useful diagnostic agents of modern times. Yet in osseus traumatisms this mode of photography is by no means free from errors and uncertainties. Except in the hands of an expert it is quite useless, and even then the shadowgraph is not infallible. No two of them are quite alike. One may exhibit a sound limb as sundered, and in another, perfect continuity where the evidence of fracture is unequivocal. With a method so full of vagaries, errors and ambiguities, we must necessarily accept with reserve its revelations, a diagnosis on which alone, in very obscure cases, is certainly not indefensible.

*The open incision* involves a surgical operation, and has been recently advocated as a means of diagnosis. It is certain that the scalpel will remove all possible doubt, but in the greater number of cases of closed fracture their recognition is possible by simple methods. Moreover, should infection with suppuration follow, unless special conditions justified it, this sequence might involve us in serious troubles. From my own very limited experience with it, however, under proper precautions, it seems to be a perfectly harmless procedure. Here before you are two patients who came into my service within the past month on whom it was employed. You will observe that one is a young man and the other a man advanced in years. The former, ten days before he entered, sustained an injury at the ankle in a brawl. He was first treated for a sprain; having no improvement he consulted another physician, who was in doubt, and who sent him to my clinic for a diagnosis. At that time the ankle and foot were swollen and edematous, the joint stiff and parts highly sensitive. An incision three inches long, four inches above the head of the fibula, readily exposed an oblique nondisplaced fracture. There was a free discharge of venous blood. The incision was immediately completely closed, and a plaster dressing applied. In the other, though only treated three weeks ago, you will observe that the incision is completely healed. He sustained an injury to the forearm six weeks since; went to a dispensary and was given a liniment, the arm was bathed and massaged. He was advised to keep up free motion in the wrist and elbow to overcome the rigidity, but this he found was impossible, because of the great pain it provoked. On a very thorough examination we believed that there was a probable fracture near the round head of the radius. Here, again, a free incision was made when another nondisplaced fracture of the radial shaft was discovered. The wound was hermetically sealed and a support applied. In both of these cases the results have been very gratifying, and it would certainly seem that the division of the tense indurated soft parts exercised a most salutary influence by relieving pressure, by local depletion and favoring reparative processes in the osseus parts, as you will find on examination, perfect consolidation of the fragments. We now turn to the last, or perhaps what should be the first step, a *consultation* with a practitioner whose knowledge is greater than our own in this type of traumatism. In all severe or doubtful fractures one should not neglect this, both for the patient's advantage, to divide the responsibility and protect ourselves. If circumstances prevent it, one may send the case to a hospital, where our responsibility ends.

### Periurethral Abscess.

The special essential point emphasized by Bernart, in regard to follicular and periurethral abscess, is to make sure of the diagnosis, for a follicular abscess may closely simulate a chancre or a gumma; he refers to occluded abscesses that do not discharge freely. He says we should try all the mechanical means available to produce an opening of the occluded duct or an absorption of the deposit, but never await the breaking of a follicular abscess, as the destruction of the tissue is greater, or it may break both externally and internally, or may rupture internally and point backward, thus favoring urinary infiltration. We should open through the urethra, if possible.—*Memphis Medical Monthly.*

